# Structures of partition protein ParA with nonspecific DNA and ParB effector reveal molecular insights into principles governing Walker-box DNA segregation

**DOI:** 10.1101/gad.296319.117

**Published:** 2017-03-01

**Authors:** Hengshan Zhang, Maria A. Schumacher

**Affiliations:** Department of Biochemistry, Duke University Medical Center, Durham, North Carolina 27710, USA

**Keywords:** ParA, Walker-box, DNA segregation, nucleoid, HDR, ParB, diffusion ratchet

## Abstract

Walker-box partition systems are ubiquitous in nature and mediate the segregation of bacterial and archaeal DNA. Well-studied plasmid Walker-box partition modules require ParA, centromere–DNA, and a centromere-binding protein, ParB. In these systems, ParA–ATP binds nucleoid DNA and uses it as a substratum to deliver ParB-attached cargo DNA, and ParB drives ParA dynamics, allowing ParA progression along the nucleoid. How ParA–ATP binds nonspecific DNA and is regulated by ParB is unclear. Also under debate is whether ParA polymerizes on DNA to mediate segregation. Here we describe structures of key ParA segregation complexes. The ParA–β,γ-imidoadenosine 5′-triphosphate (AMPPNP)–DNA structure revealed no polymers. Instead, ParA–AMPPNP dimerization creates a multifaceted DNA-binding surface, allowing it to preferentially bind high-density DNA regions (HDRs). DNA-bound ParA–AMPPNP adopts a dimer conformation distinct from the ATP sandwich dimer, optimized for DNA association. Our ParA–AMPPNP–ParB structure reveals that ParB binds at the ParA dimer interface, stabilizing the ATPase-competent ATP sandwich dimer, ultimately driving ParA DNA dissociation. Thus, the data indicate how harnessing a conformationally adaptive dimer can drive large-scale cargo movement without the requirement for polymers and suggest a segregation mechanism by which ParA–ATP dimers equilibrate to HDRs shown to be localized near cell poles of dividing chromosomes, thus mediating equipartition of attached ParB–DNA substrates.

## Abstract (precis)

Zhang et al. describe structures of key ParA segregation complexes. The data reveal how harnessing a conformationally adaptive dimer can drive large-scale cargo movement without the requirement for polymers.

The propagation of genetic material, termed DNA segregation or partition, is essential for all life and is therefore one of the most fundamental biological processes. Prokaryotic plasmid partition (*par*) systems have served as excellent models to study this fundamental process at the atomic level due to their simplicity. DNA segregation by these systems requires, minimally, a centromere site located on the DNA to be segregated; a nucleotide triphosphatase (NTPase), typically called ParA; and a centromere-binding protein (CBP), termed ParB (Gerdes et al. 2000, 2010; Surtees and Funnell 2003; Schumacher 2012; Baxter and Funnell 2014). Three main plasmid *par* systems have been identified based on NTPase sequence homology (Gerdes et al. 2010). The less abundant type II and type III systems encode actin and tubulin-like NTPases, respectively. In these systems, the NTPases form polymers to mediate DNA segregation. The actin-like polymers bind and push apart replicated DNA plasmid cargo in a process termed insertional polymerization, while tubulin-like NTPase filaments undergo treadmilling and pull CBP-bound cargo DNA to cell poles (Egelman 2003; Møller-Jensen et al. 2003; Pogliano 2004; Garner et al. 2007; Schumacher et al. 2007; Gerdes et al. 2010; Ni et al. 2010; Gayathri et al. 2012; Schumacher 2012; Bharat et al. 2015; Fink and Löwe 2015). The less well-understood type I Walker-box systems are used by bacterial and archaeal chromosomes and plasmids and hence are arguably the most ubiquitous type of partition system in nature (Gerdes et al. 2000; Schumacher et al. 2015; Barillà 2016).

A distinguishing feature of the Walker-box systems is that their ParA NTPases bind and use nonspecific nucleoid DNA (nsDNA) as a substratum to equipartition replicated DNA (Bouet et al. 2007; Castaing et al. 2008; Ringgaard et al. 2009; Vecchiarelli et al. 2010, 2012; Hwang et al. 2013). However, the molecular details by which Walker-box ParA proteins bind nsDNA and how their partner ParB CBP proteins collaborate with them to drive segregation have been controversial. Indeed, two distinct mechanisms have been proposed for Walker-box partition: a polymer-based model in which ParA proteins form filaments on nsDNA that move and direct ParB–DNA cargo (Barillà et al. 2005; Lim et al. 2005; Ebersbach et al. 2006; Bouet et al. 2007; Hatano et al. 2007; Ringgaard et al. 2009; Gerdes et al. 2010; Ptacin et al. 2010) and a nonpolymer diffusion ratchet-like mechanism in which the destabilization of ParA DNA binding by ParB establishes a ParA–ATP gradient on the nucleoid that attracts ParB–DNA cargo (Vecchiarelli et al. 2010, 2013a,b, 2014; Hwang et al. 2013). Le Gall et al. (2016) recently proposed a modified version of the diffusion ratchet model in which ParA piggybacks on the chromosome DNA.

ParA Walker-box proteins come in two main types: small ∼200- to 230-residue proteins that contain only Walker-box folds and larger proteins of ∼250–440 residues, exemplified by P1 ParA, that contain, in addition to their Walker-box regions, N-terminal winged helix–turn–helix (HTH) domains (Dunham et al. 2009). The ADP-bound forms of the larger Walker-box proteins are dimeric and bind specific operator sites with their winged HTHs to effect transcription autoregulation of their respective *par* operons (Bouet and Funnell 1999; Gerdes et al. 2000; Dunham et al. 2009). In contrast, this autoregulatory role is fulfilled by the CBP proteins in the case of the *par* systems containing small Walker-box ParA proteins and the CBP proteins in the type II and type III *par* systems (Schumacher 2012; Baxter and Funnell 2014). However, both the larger winged HTH-containing and small ParA proteins use their Walker-box domains to engage the nucleoid and use it as a track for their partition functions (Vecchiarelli et al. 2010, 2012). The ParB proteins not only bind the centromere sites on the replicated DNA but also function to trigger movement of ParA along the nucleoid substratum. Multiple ParB proteins bind cooperatively to centromere sites on the cargo DNA to form large partition complexes (Rodionov et al. 1999; Schumacher and Funnell 2005; Schumacher 2012; Graham et al. 2014; Chen et al. 2015; Funnell 2016). Data indicate that disordered typically N-terminal regions of ParB proteins displayed on the partition complexes bind their partner ParA proteins to mediate partition dynamics by stimulating ParA–ATP hydrolysis (Barillà et al. 2007; Vecchiarelli et al. 2013a; Schumacher et al. 2015; Volante and Alonso 2015). ParA must be complexed with ATP to bind DNA. Hence, ParB drives ParA off the nucleoid. ATP recomplexation by ParA allows it to also rebind DNA, permitting it to advance along the nucleoid. In the polymer model, ParB binding to ParA is postulated to cause polymer retraction with the concomitant “dragging” of ParB–DNA cargo in the polymer wake (Ringgaard et al. 2009; Gerdes et al. 2010). The diffusion ratchet model is based largely on in vitro reconstitution experiments with DNA curtains (Vecchiarelli et al. 2010, 2013a,b, 2014, 2015; Hwang et al. 2013). According to this model, ParB-stimulated ParA–ATP hydrolysis leads to the dissociation of ParA dimers into monomers with the concomitant DNA dissociation by ParA. Interestingly, biochemical data indicate that released ParA recomplexes with ATP but cannot bind DNA until a transition occurs in the ParA–ATP structure. The acquisition of the new structural state, called ParA–ATP*, leads to a time delay. Hence, ParA–ATP does not rebind in the same location and, in the new state, appears to bind more stably to nsDNA (Vecchiarelli et al. 2010). Therefore, in both models, ParB acts as not only a conduit for delivery of the cargo DNA but also a ParA effector.

While Walker-box segregation is driven by the two key molecular steps involving ParA complexation with nsDNA and its interaction with ParB, there are currently no structures available for these complexes. Therefore, how these steps are mediated and whether ParA polymers are involved are not clear. To address these questions, we obtained crystal structures of ParA–β,γ-imidoadenosine 5′-triphosphate (AMPPNP)–nsDNA and ParA–AMPNP–ParB complexes and performed complementary in vivo and biochemical analyses. The combined data support that ParA proteins do not form polymers on nsDNA. Furthermore, in vivo analyses suggest that ParA proteins interact with nucleoid DNA in a manner that does not depend on organism- or domain-specific (i.e., archaeal vs. bacterial) factors. The data also show how binding nsDNA and the ParB effector drive ParA into distinct functional dimer states that take advantage of nucleoid properties to enable transport of large molecular cargo without the requirement for polymer formation, with the ultimate result being equipartition of replicated genomic DNA.

## Results

### Crystal structure of the nonspecific ParA–AMPPNP–DNA complex

**Figure 1.**
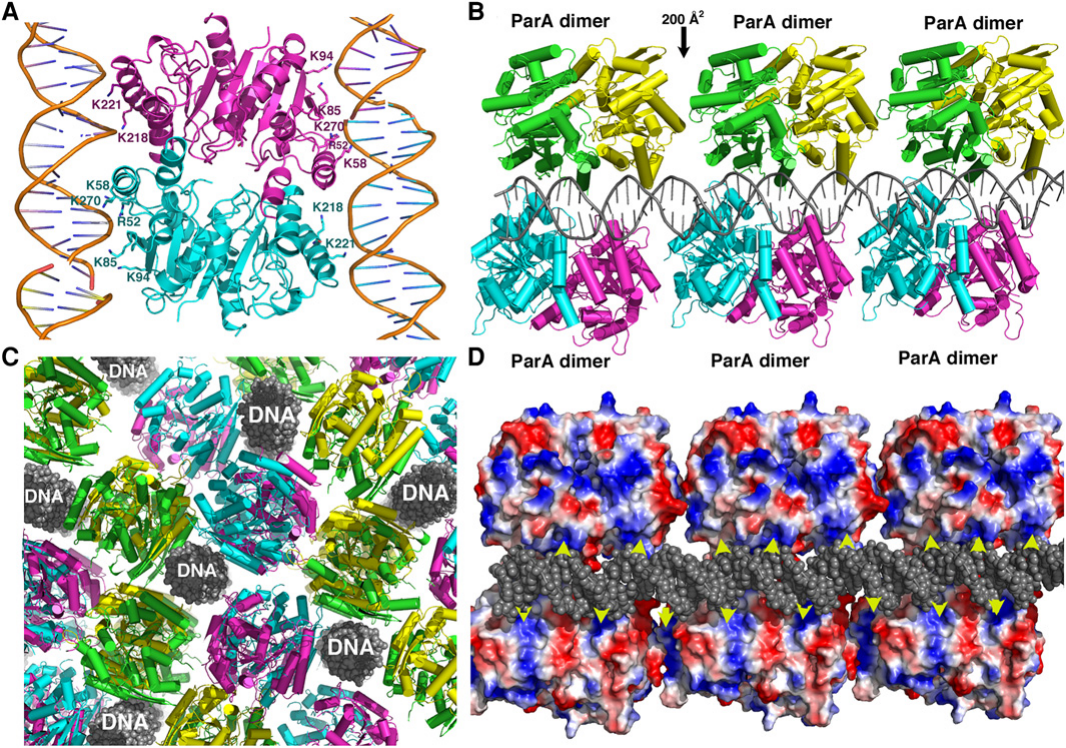
Structure of pNOB8 ParA–AMPPNP–DNA complex. (*A*) Ribbon diagram of the pNOB8 ParA–AMPPNP–DNA complex. (*B*) Packing of ParA along the DNA in the crystal reveals no close contacts between ParA dimers, indicating that it does not polymerize on DNA. (*C*) Side view of the ParA–DNA crystal packing showing how, by contacting several DNA duplexes, each ParA dimer can embed in a dense DNA substrate. (*D*) Electrostatic surface representation of the view shown in *B*, highlighting the complementary periodicity between ParA basic residues and the negatively charged DNA backbone.

A unique and essential feature of Walker-box partition is nsDNA binding by the ParA NTPases. However, how these proteins interact with nsDNA has been unknown. Notably, all Walker-box ParA proteins, including the recently characterized archaeal pNOB8 ParA, show strong structural homology (Leonard et al. 2005; Pratto et al. 2008; Dunham et al. 2009; Schumacher et al. 2012, 2015). The pNOB8 ParA protein is encoded on the pNOB8 plasmid, which is harbored in *Sulfolobus* NOB8H2 and is part of a partition cassette that also encodes a CBP and adaptor (Schumacher et al. 2015). Because obtaining structures of protein–nsDNA complexes is challenging, we carried out crystallization trials of multiple ParA proteins, including pNOB8 ParA, with DNA in the presence of ATP or the ATP analog AMPPNP to maximize chances of obtaining a ParA–DNA crystal structure (Materials and Methods). Structures were ultimately obtained of the archaeal pNOB8 ParA protein bound to DNA. pNOB8 ParA–AMPPNP–DNA and pNOB8 ParA–ATP–DNA structures were obtained to 2.95 and 3.30 Å, respectively. As the structures were essentially identical, the higher-resolution ParA–AMPPNP–DNA structure (R_work_ = 22.0% R_free_ = 24.7%) was used for analyses (Materials and Methods). In the pNOB8 ParA–AMPPNP–DNA structure, the DNA packs pseudocontinuously and is statistically disordered, consistent with data (Castaing et al. 2008; Gerdes et al. 2010; Vecchiarelli et al. 2010; Schumacher et al. 2015; Le Gall et al. 2016) showing that ParA proteins do not bind DNA specifically. However, to assess this further, crystals were obtained of a ParA–AMPPNP–DNA complex with the same DNA sequence but containing two bromo-deoxyuridines in the place of thymines (5′-U_Br_GACGCCGGCGU_Br_CA-3′ U_Br_ [5-bromouracil]). Anomalous data collected at the bromine edge for a crystal showed no peaks, consistent with ParA not binding the DNA in a sequence-specific manner. The ParA–AMPPNP–DNA structure revealed repeating units of ParA dimers bound to the pseudocontinuously packed DNA (Fig. 1A–D; Supplemental Fig. S1). Notably, only weak contacts were observed between ParA dimers. The largest buried surface area between dimers in the structure is between the adjacent DNA-bound dimers shown in Figure 1B (∼200 Å^2^) (Fig. 1C,D; Supplemental Fig. S2A,B), and only a few side chain interactions were observed between ParA molecules that surround a given DNA (Fig. 1C; Supplemental Fig. S2B). The largest buried surface area in the next dimer–dimer interface is <200 Å^2^. Thus, these dimer–dimer contacts are not likely to be physiologically relevant. Moreover, the residues mediating interactions between dimers along or surrounding the DNA are not conserved among ParA homologs (Supplemental Fig. S2A). Thus, while such contacts may enable the formation of weak ParA aggregates, these data strongly suggest that ParA does not form polymers on DNA. Indeed, given the high ParA–DNA concentrations used to generate crystals with pseudocontinuously packed DNA, one would expect that if ParA polymers form on the DNA, then they would have been observed in the structure.

The pNOB8 ParA–AMPPNP–DNA structure showed that pNOB8 ParA interacts with the DNA backbone only and uses a plethora of basic residues, including Arg52, Lys58, Lys85, Lys218, Lys221, and Lys270, to make these contacts (Fig. 1A). Notably, the basic residues in ParA that contact the DNA are arranged in a periodic manner complementary to the DNA phosphates (Fig. 1C,D). Thus, the dimer conformation adopted by ParA in the structure appears optimal for engaging these contacts. Although there are no other ParA–nsDNA structures, previous biochemical studies showed that *Bacillus subtilis* ParA (also called Soj) residue Arg189 (Arg182 in the homologous *Thermus thermophilus* ParA for which a structure is available) and residues 242–265 in the pSM19035 ParA protein are involved in nsDNA binding (Hester and Lutkenhaus 2007; Volante and Alonso 2015). Superimpositions of the *T. thermophilus* and pSM19035 ParA structures onto the pNOB8 ParA–AMPPNP–DNA structure reveals that these residues are localized next to pNOB8 ParA nsDNA-binding residues Lys218 and Lys220 (Supplemental Fig. S3). Interestingly, basic residues in pSM19035 and *T. thermophilus* ParA and Soj are also positioned similarly to pNOB8 ParA DNA-binding residues Arg52 and Lys58 (Supplemental Fig. S3).

The ParA dimer in the ParA–AMPPNP–nsDNA structure harbors a multifaceted DNA-binding surface with dispersed basic residues, which allows it to bind multiple DNA duplexes (Fig. 1C,D). To further characterize DNA binding by pNOB8 ParA, we carried out fluorescence polarization (FP) DNA-binding experiments. These studies showed that the wild-type protein bound DNA (Materials and Methods) with a *K*_d_ of ∼120 nM (Supplemental Fig. S4A). To gain insight into the binding stoichiometry of pNOB8 ParA for DNA in solution, we also used FP. In these experiments, the conditions were identical to those used in the FP binding affinity determination experiments except that the concentration of DNA was increased to 1 µM, which is ∼10-fold above the *K*_d_, thereby ensuring stoichiometric binding. Wild-type pNOB8 ParA was titrated into the binding solution, and the plot of the resulting data shows a linear increase in the observed millipolarization until saturation, after which the millipolarization values showed no increase. The inflection point occurs at a ParA dimer concentration of ∼0.5 µM, which, when divided by the concentration of DNA (1 µM), indicates a stoichiometry of approximately one ParA dimer to two DNA 14mer duplexes, consistent with the structure (Fig. 1A; Supplemental Fig. S4B).

In vitro reconstitution studies carried out on multiple Walker-box systems that used DNA curtains to mimic the nucleoid revealed that ParB–cargo DNA complexes diffused away from the DNA after stimulating ParA ATPase activity (Vecchiarelli et al. 2013a, b). This finding led the investigators to speculate that the partition process must take place in a confined region between the membrane and the nucleoid to prevent Par components from floating away. However, such confinement is unlikely to be found along the extent of the nucleoid. The multifaceted DNA-binding surface revealed in the ParA–AMPPNP–DNA structure indicates that ParA would favor interactions with three-dimensional (3D) DNA arrays, which would lead ParA molecules to become embedded within the volume of the nucleoid. This DNA-binding property is consonant with biochemical data showing that ParA–ATP engages in intersegmental transfer between DNA sites (Vecchiarelli et al. 2010) as well as more recent superresolution analyses showing that ParA proteins appeared to localize to high-density DNA regions (HDRs) within chromosomes (Marbouty et al. 2015; Le Gall et al. 2016). The multidimensional DNA-binding feature revealed in the ParA dimer from the ParA–AMPPNP–DNA structure would therefore negate the requirement for membrane–nucleoid confinement, as DNA-embedded ParA would trap its ParB–DNA cargo in the nucleoid cloud, preventing its escape into the cytosol.

### The ParA–nsDNA interaction is independent of domain/organismal-specific factors

**Figure 2.**
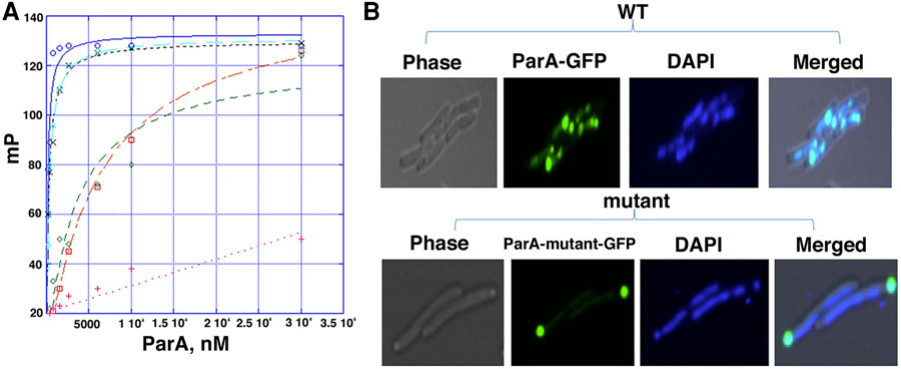
In vivo and in vitro tests of the ParA–DNA model and organism independence in ParA–nucleoid binding. (*A*) FP binding isotherms for wild-type ParA (blue), ParA(K270E) (cyan), ParA(K58E) (black), ParA(R52E-K218E) (red), ParA(R52E-K221E), and ParA(R52E-K218E-K221E-K270E) (pink). The corresponding *K*_d_s are 120 nM ± 10 nM, 300 nM ± 40 nM, 274 nM ± 44 nM, 3190 nM ± 90 nM, 5933 nM ± 1000 nM, and NB (no measurable binding), respectively. The *X*-axis and *Y*-axis indicate ParA concentration in nanomolar and millipolarization (mP) units, respectively. (*B*) Confocal microscopy examining the localization of wild-type ParA-GFP (*top*) and ParA(R52E-K218E-K221E-K270E) (*bottom*). Nucleoid DNA was stained blue with DAPI. Images at the *left* were obtained from phase contrast microscopy.

To probe the ParA–AMPPNP–DNA model in more detail, we assessed the in vitro and in vivo consequences of mutating residues shown to be involved in DNA binding in the structure. Using FP binding assays, we showed that pNOB8 ParA, like other ParA proteins, binds nsDNA only when complexed to an ATP or ATP analog; no DNA binding was observed by pNOB8 apo or ADP-bound ParA (Supplemental Fig. S4A). We next analyzed the effect of the single substitutions of residues shown by the ParA–AMPPNP–DNA structure to be important for DNA binding. ParA(K58E) and ParA(K270E) substitutions resulted in twofold to threefold reductions in DNA-binding affinity (Fig. 2A). The introduction of two mutations in ParA, such as in ParA(R52E–K218E) and ParA(R52E–K221E), caused significant (∼30-fold to 60-fold) loss in affinity, and mutating five of the basic residues shown to be involved in DNA binding by the structure [ParA(R52E–K85E–K218E–K221E–K270E)] essentially abrogated ParA's interaction with DNA (Fig. 2A).

Both archaea and bacteria harbor exposed nucleoids that are not enclosed within a nuclear envelope, perhaps explaining the evolution of nucleoid-based Walker-box systems in these organisms. To test the effects of pNOB8 ParA mutants on DNA binding in vivo, we carried out confocal microscopy studies in *Escherichia coli*. As these studies analyzed the ability of an archaeal ParA to interact with a bacterial chromosome, they also addressed the question of whether a non-membrane-encased nucleoid is all that is required for ParA DNA binding. The bacterial TP228 ParA-GFP fusion protein was used as a control and, as previously reported, localized with the *E. coli* chromosome (Supplemental Fig. S5; Ringgaard et al. 2009; McLeod et al. 2016). Colocalization was also revealed between wild-type archaeal pNOB8 ParA-GFP and the bacterial nucleoid (Fig. 2B). In contrast, pNOB8 ParA(R52E–K85E–K218E–K221E–K270E), which FP studies revealed was defective in DNA binding, localized to regions outside the nucleoid (Fig. 2B). Thus, these combined data support the ParA–DNA structural model and also reveal that an archaeal ParA can bind a bacterial nucleoid, indicating that ParA proteins need only an exposed DNA substratum for interaction and not organismal- or domain-specific (archaea vs. bacteria) factors.

### The ParA–AMPPNP–DNA structure captures the ParA–ATP* dimer state

**Figure 3.**
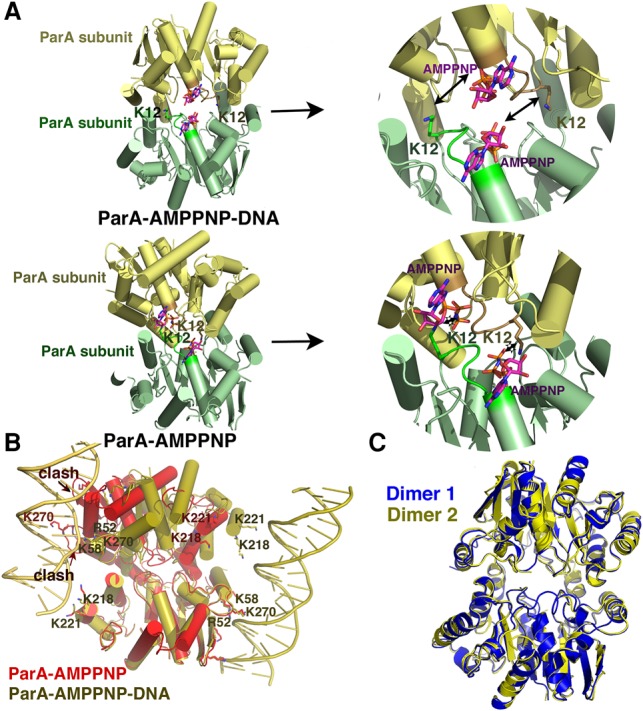
The ParA–AMPPNP–DNA structure captures the putative ParA–ATP* state. (*A*) Comparison of pNOB8 ParA–AMPPNP–DNA (*top*) and ParA–AMPPNP (*bottom*) dimers. The green subunits are in the same orientation, underscoring the different dimer conformations of the two ParA structures. ParA–AMPPNP harbors the canonical nucleotide sandwich structure with signature lysines (Lys12) inserted into the active site of the adjacent subunit (*right*, close-up view). The Lys12 in the DNA-bound form are far from the adjacent subunit's active site. (*B*) Superimposition of one subunit of the ParA–AMPPNP (red) and ParA–AMPPNP–DNA (yellow) dimers, further underscoring their different dimer states. (*C*) Overlay of the ParA dimers in the ParA–AMPPNP–DNA asymmetric unit showing that they adopt the same conformation.

Previous electron microscopy (EM) studies showing ParA polymerization in the presence of ATP or ATP analog were used as support for a polymer segregation model (Barillà et al. 2005; Lim et al. 2005; Ebersbach et al. 2006; Bouet et al. 2007). However, DNA-bound ParA–ATP is the physiologically relevant species. EM experiments on pNOB8 ParA–AMPPNP showed that, similar to other ParA proteins, pNOB8 ParA–AMPPNP forms irregular polymers in the absence of DNA, but no polymeric structures were observed in the presence of nsDNA (Supplemental Fig. S6A–C). Why ParA proteins form polymers at high concentrations in the absence of DNA is unclear, but the physiologically germane ParA species is that bound to the nucleoid, which shows no capacity to form polymers. Our data showed that this is also the case for the archaeal pNOB8 ParA protein. Thus, our combined data are not consistent with a polymer segregation mechanism and instead support diffusion ratchet-based models. One prediction from diffusion ratchet-type models is that ParA–ATP dimers undergo a conformational transition to a ParA–ATP* state, which is licensed for DNA binding (Vecchiarelli et al. 2010, 2013a). This conversion explains an observed time delay that prevents the rapid DNA rebinding by ParA–ATP (Vecchiarelli et al. 2010). While the existence of this state was revealed biochemically, the 3D structural details of the distinct ParA–ATP* state have been elusive (Vecchiarelli et al. 2010). In ParA–ATP-bound structures, the so-called signature lysines from one subunit (Lys12 in pNOB8 ParA) insert into the active site of the adjacent subunit, contacting its ATP phosphates, creating the so-called nucleotide sandwich dimer form of ParA (Leonard et al. 2005; Schumacher et al. 2012). This interaction stimulates ATP hydrolysis and hence ParA's dissociation from DNA. Strikingly, however, comparison of the ParA–AMPPNP–DNA and ParA–AMPPNP structures revealed that, although both of the surface areas buried in the two dimers are substantial (the DNA-bound ParA–AMPPNP dimer buries 1858 Å^2^ compared with 1861 Å^2^ for the ParA–AMPPNP dimer), indicating that these are physiologically relevant dimers, the ParA dimer in the ParA–AMPPNP–DNA structure is structurally distinct from the canonical nucleotide sandwich dimer conformation (Fig. 3A,B). Specifically, there is an ∼38° rotation of one subunit relative to the other in the ParA–AMPPNP–DNA complex compared with the nucleotide sandwich dimer form (Fig. 3B). Notably, this dimer transition exposes ParA basic residues optimally for DNA binding (Figs. 1B, 3B). Thus, the ParA dimer in the ParA–AMPPNP–DNA structure appears to reflect a conformation consistent with the proposed ParA–ATP* state (Vecchiarelli et al. 2010). Importantly, the fact that the two dimers in the ParA–AMPPNP–DNA asymmetric unit adopt the same structure indicates that this dimer conformation is not a crystallographic artifact (Fig. 3C).

Another result of the conformational switch in the ParA–AMPPNP–DNA structure is that the Lys12 side chains are displaced ∼8 Å from the neighboring subunit's ATP γ-phosphate (cf. 2.5–3.0 Å in ParA–AMPPNP) (Fig. 3A). In this dimer form, the ATPs are highly solvent-exposed and appear to be less tightly complexed. This finding is supported by the poor electron density observed for the nucleotides in this structure (Materials and Methods; Supplemental Fig. S7). This finding appears consistent with previous data indicating an increased ATP off rate for the ParA–ATP* state, which might also explain data indicating that the ParA–ATP* state exhibits slightly increased ATPase activity (Vecchiarelli et al. 2010). Interestingly, while this suggests that the DNA-bound ParA dimer form may not be as tightly complexed with ATP, this dimer notably does not depend on ATP cross-contacts between subunits, which may impart DNA-binding stability to this ParA state.

### The ParA–AMPPNP–ParB structure reveals the basis for ParB ATPase activation of ParA

Cyclical nucleoid binding and unbinding by ParA ultimately creates a moving wave of ParA molecules that attract the ParB–DNA cargo to follow in their wake. Data have shown that ParB somehow mediates these dynamics by stimulating ParA's ATPase activity and dissociation from nsDNA (Bouet et al. 2007; Dunham et al. 2009; Vecchiarelli et al. 2010; Schumacher et al. 2012, 2015). How ParB proteins perform this function is unclear, but data show that ParA proteins bind to flexible regions of their partner ParB proteins (Surtees and Funnell 1999; Barillà and Hayes 2003; Golovanov et al. 2003; Weihofen et al. 2006; Ringgaard et al. 2009; Schumacher et al. 2015). These flexible regions or arms are attached via a linker to folded DNA-binding domains consisting of either HTH or ribbon–helix–helix motifs (Schumacher 2012). Understanding this essential step of ParB binding to ParA–AMPPNP and how it drives the dynamics of the system necessitates structural information. Hence, we carried out crystallization trials of multiple ParA–AMPPNP–ParB complexes (Materials and Methods). Crystals were obtained of the complex formed between the TP228 plasmid ParA and ParB proteins (also called ParF and ParG) in the presence of AMPPNP (Table 1). TP228 is a conjugative plasmid harbored in bacteria; it was originally identified in *Salmonella newport*. This plasmid is clinically important, as it confers resistance to a range of antibiotics (kanamycin, neomycin, spectinomycin, streptomycin, sulphonamides, and tetracycline) as well as mercuric ions (Couturier et al. 1988). TP228 can replicate in low copy number in *E. coli*, and its retention depends on the type I Walker-box cassette that includes the small 206-residue Walker-box ParA protein and the 76-residue ParB protein, which is composed of an N-terminal disordered region that binds ParA followed by a ribbon–helix–helix DNA-binding motif (Golovanov et al. 2003; Schumacher et al. 2012).

**Table 1.**
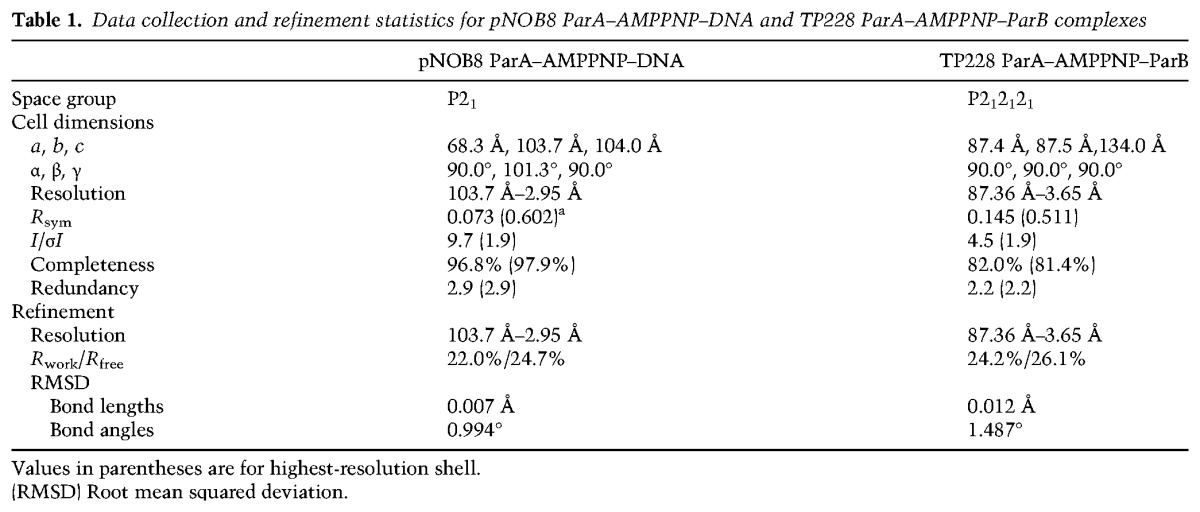
Data collection and refinement statistics for pNOB8 ParA–AMPPNP–DNA and TP228 ParA–AMPPNP–ParB complexes

**Figure 4.**
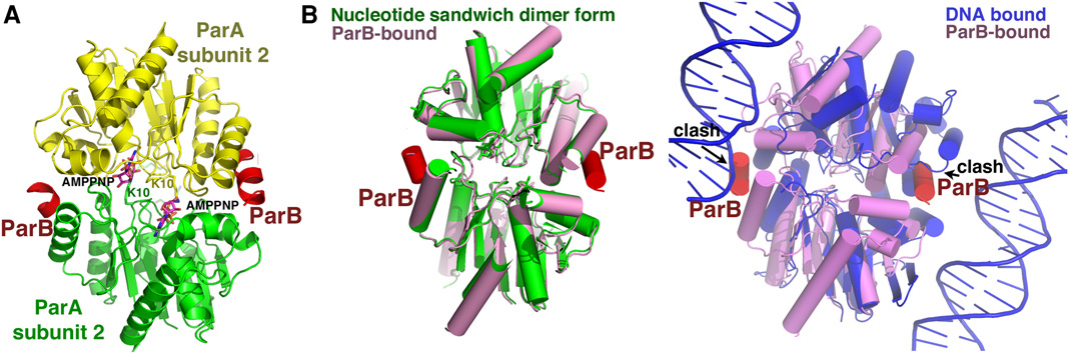
The TP228 ParA–AMPPNP–ParB structure adopts the nucleotide sandwich dimer conformation. (*A*) Overall TP228 ParA–AMPPNP–ParB structure. ParB (red) folds into a helix upon ParA binding and interacts in the ParA dimer (ParA subunits are colored green and yellow) interface, stabilizing the nucleotide sandwich dimer form. Signature lysines, Lys10, and AMPPNP are shown as sticks. (*B*, *bottom left*) Superimposition of one subunit from the TP228 ParA–AMPPNP–ParB structure (pink) onto the TP228 ParA–AMPPNP structure (green) (4E07). The ParB helices from the ParA–AMPPNP–ParB structure are included and colored red. The AMPPNP molecules overlay well between the structures and are shown as sticks. Like ParA–AMPPNP, the ParB-bound ParA–AMPPNP adopts the canonical nucleotide sandwich dimer conformation observed in other ParA–ATP analog structures; the ParA dimers in the structures superimpose with a root mean squared deviation (RMSD) of 0.7 Å for 410 Cα atoms. (*Right*) Superimposition of the “bottom” TP228 ParA subunit of ParA–AMPPNP–ParB (pink) onto that of the pNOB8 ParA–AMPPNP–DNA (blue) structure. The resulting RMSD is 1.9 Å for 189 Cα atoms. The ParB helices that bind TP228 ParA are red. The ParB helices would clash with ParA and DNA if they were to adopt the DNA-bound state.

Crystals of the TP228 ParA–AMPPNP–ParB complex took 6 mo to grow, and the structure was solved to 3.65 Å. The structure revealed density for only ∼15 N-terminal ParB residues, consistent with the subsequent finding that it had degraded during the extended crystallization process. In the structure, the ParB fragments dock as helices within the crevice at the ParA dimer interface (Fig. 4A). The formation of the ParB–ParA complex buries ∼450 Å^2^ of protein surface from solvent, consistent with data indicating that the ParA–ParB interaction is weak (Vecchiarelli et al. 2010; Volante and Alonso 2015). Strikingly, the ParB helices interact specifically with the ParA subunit arrangement found in the nucleotide sandwich dimer state; comparison of the ParA–ParB and ParA–DNA structures shows that ParB cannot dock in the dimer interface of the conformation that ParA adopts when bound to DNA without clash (Fig. 4B). Thus, the structure indicates that ParB binding stabilizes the ParA nucleotide sandwich dimer conformation and not the DNA-binding state (Fig. 4B).

**Figure 5.**
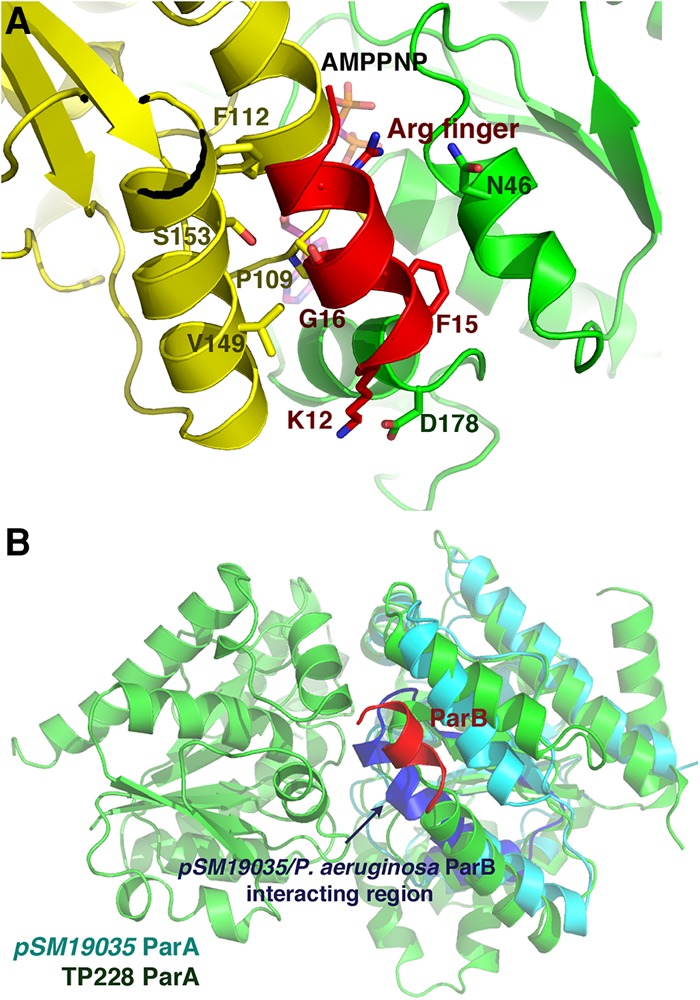
ParB-binding site on ParA. (*A*) Close-up view of the ParB–ParA interaction. Shown is one ParB N-terminal helix docked within the cavity created in the ParA nucleotide dimer state. One ParA subunit is yellow, and the other is green. The AMPPNP is shown as sticks. The binding of ParB places Arg19 within proximity to the ParA active site such that it could act as an arginine finger. (*B*) Overlay of a subunit of pSM19035 ParA (also called δ) onto one ParA subunit in the TP228 ParA–AMPPNP–ParA complex. TP228 ParA is colored green, and the TP228 ParB helix is red. pSM19035 ParA is colored cyan with the exception of pSM19035 ParA residues 88–119, which are colored blue. These residues and the corresponding residues in the *Pseudomonas aeruginosa* ParA were shown to interact with pSM19035 ParB and the *P. aeruginosa* ParB, respectively (Bartosik et al. 2014; Volante and Alonso 2015), and overlap with the TP228 ParB-binding site in TP228 ParA.

The region of TP228 ParB that binds ParA was mapped previously to an N-terminal domain (residues 15–23) in the protein, which is disordered in the absence of ParA (Barillà et al. 2007). This analysis also showed that Arg19 stimulates the ATPase activity of ParA, leading the investigators to suggest that it may function as an arginine finger (Barillà et al. 2007). Although the ParA–AMPPNP–ParB structure is low resolution, the best fit to the ParB helical density places ParB hydrophobic residues pointing into the ParA dimer interface with Gly16 juxtaposed next to ParA residues Val1149 and Pro109 (Fig. 5A). Any residue other than a glycine at position 16 would clash with these ParA residues. While the Arg19 side chain position is equivocal due to the low resolution of the current structure, this fit positions ParB Arg19 proximal to the AMPPNP γ-phosphate in the ParA active site, suggesting that it could aid in the stabilization of the transition state in which the γ-phosphate becomes partially hydrolyzed (Fig. 5A). We conclude from the ParA–AMPPNP–ParB structure that ParB drives the dynamics of Walker-box segregation by stabilizing the ParA nucleotide sandwich dimer form, which appears nonoptimal for DNA binding and stimulating its ATP hydrolysis.

Previous biochemical studies identified regions in ParB proteins that are involved in binding to their partner ParA proteins (Bartosik et al. 2014; Volante and Alonso 2015). Work by Volante and Alonso (2015) revealed that pSM19035 plasmid ParB (also called ω) interacts with residues 88–119 in its partner, ParA (also called δ). Comparison of the pNOB8 and pSM19035 ParA structures shows that residues 88–119 of the pSM19035 protein map to the ParB-binding site observed in the TP228 ParA–AMPPNP–ParB structure (Fig. 5B). Although a structure is not available for the *Pseudomonas aeruginosa* chromosomal ParA, mutagenesis analyses revealed that residues corresponding to pSM19035 ParA residues 89–105 mediate ParB binding by this protein (Fig. 5B; Bartosik et al. 2014). Thus, these data support a shared mechanism of ParA–ParB binding by both plasmid and chromosomal partition proteins, which further indicates a conserved mode of dynamics for Walker-box segregation systems. Interestingly, the ParB–ParA complex shows similarity to the structure of MinE complexed with the Walker-box protein MinD, which functions in cell division site placement (Park et al. 2011). MinE does not appear to use an arginine finger. However, similar to ParB, MinE stimulates the ATPase activity of MinD and inserts a helix into its dimer interface. Therefore, this suggests that helical insertion by an effector protein into the Walker-box dimer interface may be a conserved mechanism for regulating Walker-box proteins.

## Discussion

**Figure 6.**
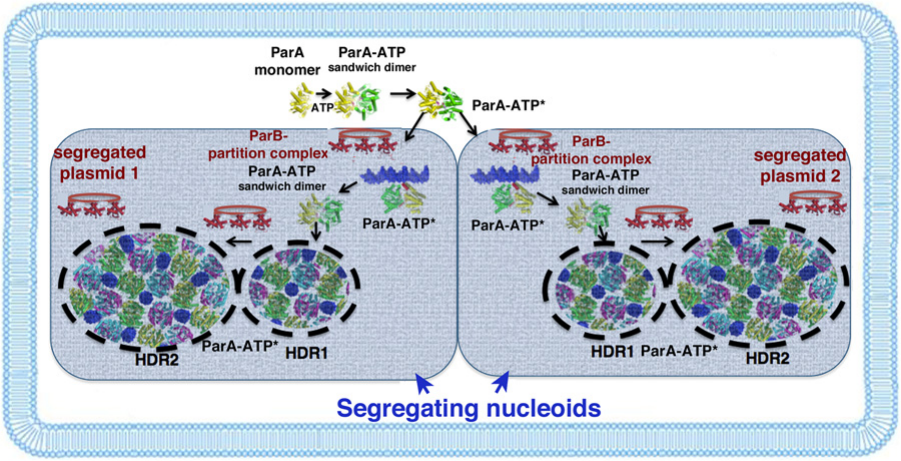
Molecular model for Walker-box segregation. Schematic showing proposed molecular steps in Walker-box segregation. In the first step, apo ParA molecules bind ATP to form the nucleotide sandwich dimer. In the presence of nucleoid DNA, the ParA–ATP structure transitions to the ParA–ATP* form, which engages nsDNA and becomes embedded within the nucleoid. Binding of the ParB–cargo DNA to ParA stabilizes the nucleotide sandwich dimer state and stimulates ATP hydrolysis. ParA dissociates, rebinds ATP, forms ParA–ATP*, and then rebinds DNA, equilibrating to HDRs. These steps continue until ParA clusters eventually equilibrate to the primary HDRs near the cell poles of the segregating host nucleoids, leading to equidistribution of the replicated plasmids to dividing cells.

Walker-box DNA segregation systems are used by both bacterial plasmids and chromosomes and more recently were identified in archaeal plasmids and chromosomes (Gerdes et al. 2000; Kalliomaa-Sanford et al. 2012; Schumacher et al. 2015; Barillà 2016). Thus, these partition modules are arguably among the most ubiquitous types of partition system in nature. A distinguishing feature of these systems is the use of a nucleoid as a substratum to transport replicated DNA cargo. However, despite their prevalence, the molecular mechanisms that drive Walker-box *par* systems have been unclear. In particular, it has been under debate whether ParA forms polymers to mediate partition. In addition, the molecular mechanism by which the ParB protein, which also serves as the cargo carrier, stimulates the ATPase activity of ParA to drive its dynamical movement is not known. Here we describe the first structures of ParA–AMPPNP–nsDNA and ParA–AMPPNP–ParB complexes. The resultant structural findings provide key insight into these partition steps that, when combined with previous biochemical in vitro reconstitution (Vecchiarelli et al. 2013a,b, 2014) and superresolution (Marbouty et al. 2015; Le Gall et al. 2016) studies, support a diffusion ratchet-like mechanism. Central to this model is our structural finding that, in the presence of DNA, ParA–ATP assumes a dimer conformation favorable for DNA binding, consistent with the predicted ParA–ATP* state (Vecchiarelli et al. 2010). In addition, the multifaceted mode of ParA DNA binding revealed in the structure would be predicted to drive both ParA and its ParB-attached DNA cargo to HDRs of the nucleoid. Binding within the nucleoid would prevent ParA and Par–cargo DNA from floating away into the cytosol. A recent study suggested that the elastic properties of the nucleoid itself might play a role in ParA transport (Lim et al. 2014). However, ParA does not display normal dynamics on the nucleoid in the absence of ParB; ParB is required for accurate ParA-mediated movement along the nucleoid (Le Gall et al. 2016). Indeed, ParA must unbind from DNA to allow its progression along the nucleoid substratum, and this function is driven by ParB. Our ParA–AMPPNP–ParB structure suggests that ParB drives the dynamics of the system by binding ParA and stabilizing its nucleotide sandwich dimer state. Thus, these molecular steps set up a catch and release process in which cycles of ParA binding and unbinding from DNA control movement of the system along the nucleoid (Fig. 6). The ParB–ParA interaction, albeit weak, ensures that the ParB–cargo DNA follows the progressing ParA wave.

However, a central question is how this system would lead to the equal segregation of ParB–cargo to dividing cells. Recent 3D superresolution studies revealed that the most prominent bacterial HDRs are located at the ends of the segregating nucleoids near the cell poles (Marbouty et al. 2015). Thus, these data suggest a molecular segregation mechanism in which ParA and its ParB–DNA cargo would equilibrate to these prominent HDRs either near the surface of the nucleoid or within the nucleoid (HDR2 in Fig. 6), ultimately resulting in the piggybacking of the Par proteins and attached DNA cargo with the nucleoid and their equipartitioning to dividing cells. Superresolution analyses support that this mechanism takes place within the nucleoid (Le Gall et al. 2016); however, more data will be required to test whether this is the case for all Walker-box systems. Finally, these combined data suggest how a conformationally adaptive protein dimer, by assuming distinct states that dictate different functions, can power large-scale macromolecular cargo movement without the need for polymerization.

## Materials and methods

### Protein expression and purification of pNOB8 ParA

pNOB8 ParA expressed as a his-tagged protein with either N-terminal or C-terminal tags was insoluble at all tested temperatures of protein induction. Hence, a construct was designed in which an MBP tag was encoded N-terminal to the pNOB8 *parA* gene followed by a tobacco etch virus (TEV) protease cleavage site. A gene encoding the designed MBP–ParA construct codon-optimized for *E. coli* expression was purchased from Genscript and cloned into the pET15b vector using NdeI and BamHI sites, which also generated a his tag N-terminal to MBP. The construct encoding his_6_-MBP–ParA was transformed into C41(DE3) cells. For protein expression, the *his_6_-MBP–parA*-encoding cells were grown to an OD_600_ of 0.6–0.8 and induced with 1 mM isopropyl β-D-1-thio-galactopyranoside (IPTG) overnight at 15°C. Cells were lysed in buffer A (25 mM Tris at pH 7.5, 300 mM NaCl, 5% glycerol, 1 mM β-mercaptoethanol [BME]) using a microfluidizer. Cell debris was removed by centrifugation at 17,000 rpm. The his_6_-MBP–ParA-containing lysate was loaded onto a Ni-NTA column, and the column was washed extensively with increasing concentrations of imidazole in buffer A. The protein was eluted with 0.1–1 M imidazole and was >95% pure at this step. The protein was next dialyzed into buffer A to remove the imidazole, and the his_6_-MBP tag was cleaved by incubating the protein with TEV overnight. The solution was then loaded onto a Ni-NTA column, and the flow-through and wash, which contained tag-free ParA, were collected, while the his_6_-MBP was retained on the Ni-NTA resin. ParA mutants were expressed as his_6_-MBP–ParA fusions and purified using the same steps as the wild-type MBP–ParA fusion. For crystallization and biochemical assays, proteins were concentrated using 30-kDa molecular weight centricon concentrators.

### Crystallization and structure determination of a pNOB8 ParA–DNA complex

In attempts to obtain a ParA–AMPPNP–DNA structure, multiple ParA proteins, including P7 ParA, P1 ParA, TP228 ParA, and pNOB8 ParA, were used in crystallization trials with AMPPNP/ATP and various DNA fragments. Crystals were ultimately obtained of the tag-free pNOB8 ParA protein in complex with 2 mM ATP (or AMPPNP), 1 mM MgCl_2_, and a 14mer DNA duplex (top strand: 5′-TGACGCCGGCGTCA-3′). The same crystals were obtained with either ATP or AMPPNP by mixing the protein–ATP or AMPPNP–DNA solution 1:1 with 20% PEG 4000, 10% isopropanol, and 0.1 M citrate (pH 5.5). The crystals were obtained at room temperature and took 1–2 d to grow and 1–2 wk to reach their full size. The crystals were cryoprotected straight from the drop, and data were collected at 100 K at Advanced Light Source (ALS) beamlines 5.0.2 and 8.3.1. The data sets were processed with MOSFLM, and the structures were solved by molecular replacement (MR) using the pNOB8 ParA–AMPPNP structure (Protein Data Bank ID 5K5Z) as a search model. The crystallographic asymmetric unit (ASU) contained two ParA dimers and one and a half DNA duplexes. Data were collected to 2.95 and 3.3 Å for the ParA–AMPPNP–DNA and ParA–ATP–DNA complexes. The structures were essentially identical. Hence, the high-resolution ParA–AMPPNP–DNA structure was used for analyses. Anomalous data collected at the bromine edge for a crystal containing bromo-uridine substitutions in the bound DNA revealed no anomalous peaks (even in *F*_o_–*F*_c_ maps contoured at <2 σ), indicating that the DNA is disordered (not specifically bound) in the crystal. Unlike the ParA–AMPPNP structure, where the AMPPNP density is clear for the entire nucleotide (stabilized by cross-contacts between the signature lysine and the adjacent subunit AMPPNP), the AMPPNP density in the ParA–AMPPNP–DNA structure is less well resolved. In this structure, the density is clear only for the phosphate moieties (Supplemental Fig. S7). The ParA–AMPPNP–DNA structure was refined in Phenix (Adams et al. 2010), and validation was performed using MolProbity (Chen et al. 2010). The final ParA–AMPPNP–DNA structure had a MolProbity score of 2.52, placing it in the 93rd percentile of structures of comparable resolution. There were no Ramachandran outliers, and 91.7% of residues were in the most favored region of the Ramachandran plot. For data collection and final refinement statistics, see Table 1.

### Crystallization, data collection, and structure determination of the TP228 ParA–ParB complex

In efforts to obtain a ParA–AMPPNP–ParB structure, several ParA and ParB proteins were purified and used in crystallization trials with ATP or AMPPNP. Specifically, P1, P7, and TP228 Par proteins were purified and mixed in a 1:1 molar ratio (ParA:ParB) in the presence of 2 mM AMPPNP or ATP and 1 mM MgCl_2_ and used in crystallization trials with multiple commercial and in-house sparse matrix crystal screens. Crystals were obtained only for the TP228 ParA–AMPPNP–ParB complex. TP228 ParA (also called ParF) and ParB (also called ParG) were expressed as his-tagged proteins as described previously (Barillà and Hayes 2003) and then mixed 1:1 with 2 mM AMPPNP and 1 mM MgCl_2_. Crystals were obtained at room temperature by mixing the ParA–AMPPNP–ParB complex 1:1 with a crystallization reagent consisting of 25% PEG 20000, 0.1 M MgCl_2_,and 0.1 M Tris (pH 8.5). Crystals took 6 mo to grow, and subsequent mass spectrometry of the sample revealed that the ParB protein had degraded. For data collection, the crystals were cryopreserved straight from the drop, and data were collected at ALS beamline 8.3.1. The crystals were extremely thin, resulting in an anisotropic diffraction pattern with high mosaicity (>1.5). The data were processed with MOSFLM, and, although the diffraction extended beyond 3.5 Å, the usable data went to a maximum resolution of ∼3.7 Å. The structure was solved by MR using the TP228 ParA–AMPPNP dimer structure (4E07) as a search model. Following rigid body and an initial round of XYZ refinement in the crystallography and nuclear magnetic resonance (NMR) system program (Brunger et al. 1998), helical density was evident for the ParB subunits. The ParB residues were placed, and final refinement was carried out in Phenix (Adams et al. 2010). There were two ParA dimer–ParB complexes in the crystallographic asymmetric unit, and both ParA dimers adopted the canonical nucleotide sandwich dimer conformation, with the signature lysines poised for catalysis. The final structure had a MolProbity score of 3.1, placing it in the 84th percentile of structures of comparable resolution. There were no Ramachandran outliers, and 88.1% of residues were in the most favored region of the Ramachandran plot. For data collection and final refinement statistics, see Table 1.

### EM

ParA at a concentration of 5 µM in a buffer consisting of 25 mM Tris (pH 7.5), 100 mM NaCl, 1 mM MgCl_2_, and 2 mM AMPPNP (2 mM ATP produced the same results) was used in EM analyses. To assess the effects of DNA, 5 µM ParA in the same buffer in the presence of 2 mM AMPPNP and 10 µM dsDNA (top strand: TGACGCCGGCGTCA) were mixed and imaged as for the other samples. For negative staining, grids covered with a thin carbon film were made hydrophilic by exposure to UV light and ozone using a Spectroline 11SC-1 Pencil shortwave UV lamp (Fisher Scientific, catalog no. 11-992-30) and UVP Pen-Ray lamp power supply (Fisher Scientific, catalog no. UVP99 0055 01). The grids were treated for 45 min and negatively stained using three drops of 2% uranyl acetate. Images were collected on a Philips EM420 equipped with a CCD camera.

### FP binding studies

FP-based binding assays were carried out in buffer consisting of 100 mM NaCl and 25 mM Tris-HCl (pH 7.5). Wild-type protein was analyzed in its apo-, ADP-, and ATP (AMPPNP)-bound forms, and mutant ParA proteins in the presence of ATP/AMPPNP were titrated into the binding buffer containing 1 nM DNA that contained a 5′ fluorescein label with the sequence 5′-TGACGCCGGCGTCA-3′ until saturation. The data were plotted and fit using KaleidaGraph. All analyses were conducted in triplicate. To assess the binding stoichiometry of ParA for the 14mer duplex DNA, the buffer and conditions were identical to those used in the FP binding affinity determination experiments except that the concentration of DNA was increased to 1 µM, which is ∼10-fold above the *K*_d_ (by using a solution containing 1 nM F-DNA and 0.999 µM nonfluorseceniated DNA). Wild-type pNOB8 ParA was titrated into the binding solution, and the graph of the resulting data shows a linear increase in the observed millipolarization until saturation, after which the millipolarization values showed no increase. The inflection point occurs at a ParA dimer concentration of ∼0.5 µM, which, when divided by the concentration of DNA (1 µM), indicates a stoichiometry of approximately one ParA dimer to two DNA duplexes.

### Confocal microscopy

Wild-type TP228 ParA-GFP, wild-type pNOB8 ParA-GFP, and pNOB8 ParA(R52E–K85E–K218E–K221E–K270E)-GFP were transformed into C41 (DE3) cells. Colonies expressing the transformants were grown in 2 mL of LB medium supplemented with ampicillin at 100 mg/mL overnight at 37°C with shaking at 200 rpm. One milliliter of induction culture was then made by combining 100 mL of the overnight culture with 0.9 mL of LB medium with the antibiotic plus 1.0 mM IPTG. After induction for 3 h at 37°C with shaking at the same speed, the cells were stained with DAPI for 5 min at room temperature (the staining mixture was made by combining 250 mL of the induced cells and 1.0 mL of the 1 mg/μL DAPI solution), placed on 1.2% agarose LB pads, and sealed using a gene frame and a coverslip. Confocal microscopy was performed using an inverted Zeiss 780 laser-scanning confocal microscope.

X-ray crystallographic coordinates and structure factors have been deposited in the Protein Data Bank under accession codes 5U1J and 5U1G.

## Supplementary Material

Supplemental Material
