# Supplementary material for: Structures of partition protein ParA with nonspecific DNA and ParB effector reveal molecular insights into principles governing Walker-box DNA segregation
Source: Genes Dev. 2017 Mar 1;31(5):481–92. doi: 10.1101/gad.296319.117 (PMC5393062; doi:10.1101/gad.296319.117)
Supplement: Supplemental Material [file supp_31_5_481__index.html]

Structures of partition protein ParA with nonspecific DNA and ParB effector reveal molecular insights into principles governing Walker-box DNA segregation — Supplemental Material 

# Structures of partition protein ParA with nonspecific DNA and ParB effector reveal molecular insights into principles governing Walker-box DNA segregation

## Supplemental Material

- Supplemental\_Figures.pdf
