# Supplementary material for: Structures of partition protein ParA with nonspecific DNA and ParB effector reveal molecular insights into principles governing Walker-box DNA segregation
Source: Genes Dev. 2017 Mar 1;31(5):481–92. doi: 10.1101/gad.296319.117 (PMC5393062; doi:10.1101/gad.296319.117)
Supplement: Supplemental Material [file supp_gad.296319.117_Supplemental_Figures.pdf]

SUPPLEMENTAL MATERIAL

Structures of partition protein ParA with nonspecific DNA and ParB effector reveal molecular insights into principles governing Walker-box DNA segregation

Hengshan Zhang & Maria A. Schumacher*

Author affiliation:

Department of Biochemistry, 307 Research Dr., Box 3711, Duke University Medical Center, Durham NC, 27710, USA

**Corresponding author:*

Maria A. Schumacher

Maria.Schumacher@Duke.edu

(919) 684-9468

Room 243A Nanaline Duke Bldg, 307 Research Dr., Box 3711, Durham, NC 27710

Contents of Supplemental Material

Figures S1 to S7

Figure S1

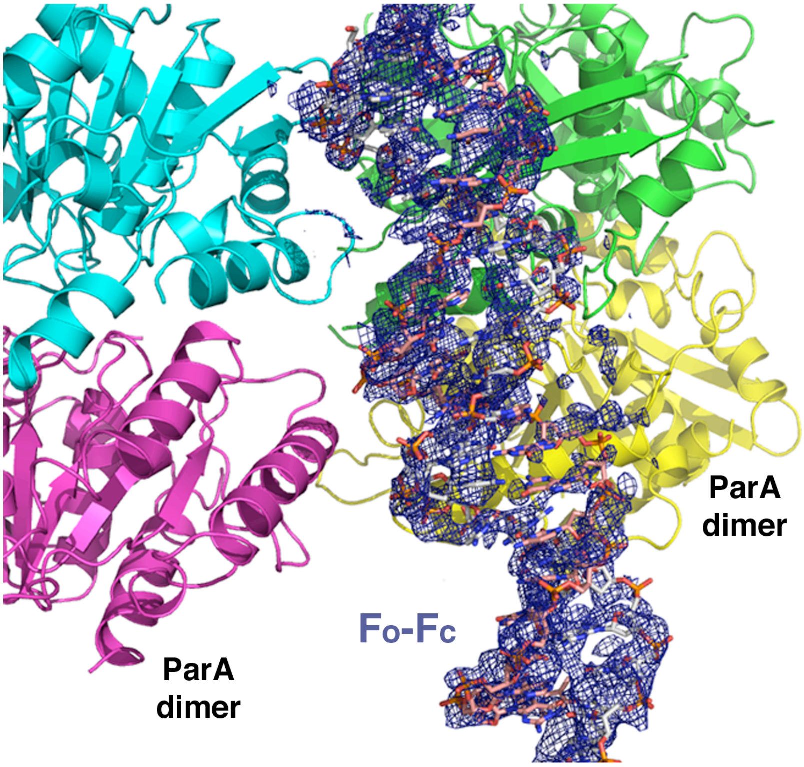

Figure S1. F_o-F_c electron density map (blue mesh) contoured at 2.7 σ, calculated before addition of the DNA to the ParA-AMPPNP-DNA structure.

Figure S2

A

```
                                                        Walker A
pNOB8 ParA    ----------------------------------MIAKVITIHNFKGGVGKTTTTAIIAMGLGAMGKRVLLIDFDAQMSLTQI   49
pSM19035 δ    MIQYYYTKKEWGVVMEKEELKILEELRRILSNKNEAIVILNNYFKGGVGKSKLSTMFAYLTDKLNLKVLMIDKDLQATLTKD   82
Soj           ------------------------------MLRAKVRRIALANQ-KGGVGKTTTAINLAAYLARLGKRVLLVDLDPQGNATSG   52
TP228 ParA    ------------------------------------MKVISFLNPKGGSGKTTAVINIATALSRSGYNIAVVDTDPQMSLTNW   47
                                                           ** ** *     *             * * *   *

             DNA binding region
pNOB8 ParA    FVREEDRLKILESSHDVTQDKSAFALLR-TME-PARIKFFHEGKGVKFGIDVIPGSYMSIFKLMFEGYIP--IQ---SEWNI   123
pSM19035 δ    LAKTFKVELPR---------VNFYEGLKNGNLASSIV’HLTD-------NLDLIPGTFDLM-LL---PKLTRSWTFENESRLL 144
Soj           LGVRAER---------------GVYHLLQGEPLEGLVHPVD-------GFHLLPAT-PDLVGAT---VEL-----AGAPTAL   103
TP228 ParA    S-KAGK-----------------------------------------AAFDVFTAAS---------------------EKDV   73

                      Walker B
pNOB8 ParA    LRMLDLYRDQYDYILIDTAPSDTVTIKPILRASHYLLIPEDGTPEAFTAMRIFLNEALPKYILPRPEGGFYKYPRILGVILT   205
pSM19035 δ    ATLLAPLKSDYDLIIIDTVPTPSVYTNNAIVASDYVMIPLQAEEESTNNIQNYISYLIDLQEQFNPGLDMI------GFVPY   220
Soj           REAL-RDE-GYDLVLLDAPPSLSPLTLNALAAAEGVVVPVQAEYYALEGVAGLLATLEEVRAGLNPRLRLL------GILVT   177
TP228 ParA    YGIRKDLA-DYDFAIVDGAGSLSVITSAAVMVSDLVIIPVTPSPLDFSAAGSVVTVLEAQA-------YSRKV--EARFLIT   138
                        **    *                     *

                 DNA binding region                                    DNA binding region
pNOB8 ParA    RVRRN---STAILMKHNKILEEELSNSELKDHVIYPPYFGADKDNPEDYILSSRKEYLSDLIWRDEKRAPISEVFDKLFLVD   284
pSM19035 δ    LVDTDSATIKSNLEELYKQHK------------EDNLVFQ-------NIIKRSNKVSTWS------KNGITEHK--------   269
Soj           MYDG-RTLLAQQVEAQLRAHFG-------------EKVFW-------TVIPRNVRLAEAP---SFGKTIA-QHAPTS-----   229
TP228 ParA    RKIEMATMLNV-LKESIKDTGV--------------KAFR-------TAITQR---------QVYVKSILDGDSVF------   183
                                                    *          *

pNOB8 ParA    DDKV-QKDLYAFFSKVFTEIPKEVVRRVENDQ---   315
pSM19035 δ    ------GYDKKVLSMYKNVFFEMLERIIQLENEKE   298
Soj           -----------PGAHAYRRLAEEVMARVQEA----   249
TP228 ParA    ------ESSDGAAKGEIEILTKEIVRIFE------   206
```

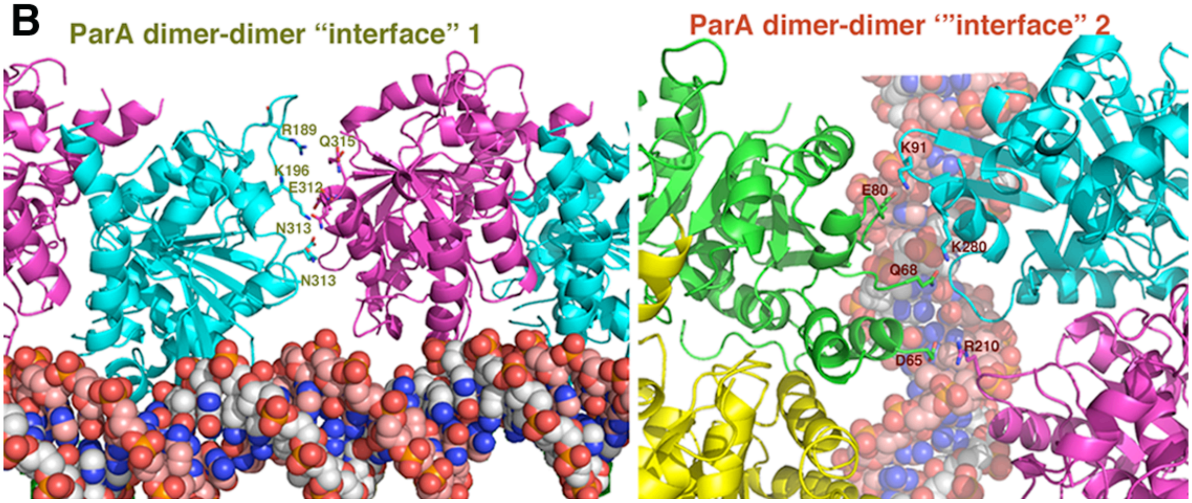

Figure S2. ParA sequence alignment and ParA dimer-dimer analysis in the ParA-AMPPNP-DNA structure. (***A***) Structure based sequence alignment of homologous ParA proteins for which structures have been obtained. Highlighted in green are the conserved Walker A and Walker B motifs that are involved in ATP binding and hydrolysis. Residues colored in cyan correspond to basic residues that contact DNA in the pNOB8 ParA-AMPPNP-DNA structure. Note, aside from the Walker motifs, these “DNA binding regions” contain similarly positioned basic residues in these homologs (also colored cyan). Colored dark yellow and red are residues involved in weak ParA dimer-dimer contacts as shown in *B*. (***B***) Analysis of ParA dimer-dimer contacts in the crystal shows that only two “interface” regions have more than 3-4 residues mediating contacts. These “interfaces” are shown in the left and right panels. The dimer-dimer contacts shown in the left panel bury the most surface area (200 Å^2) while the contacts between dimers in the right panel bury < 200 Å^2. Note, residues in these contact regions are not conserved in other ParA proteins (as seen in *A*).

Figure S3

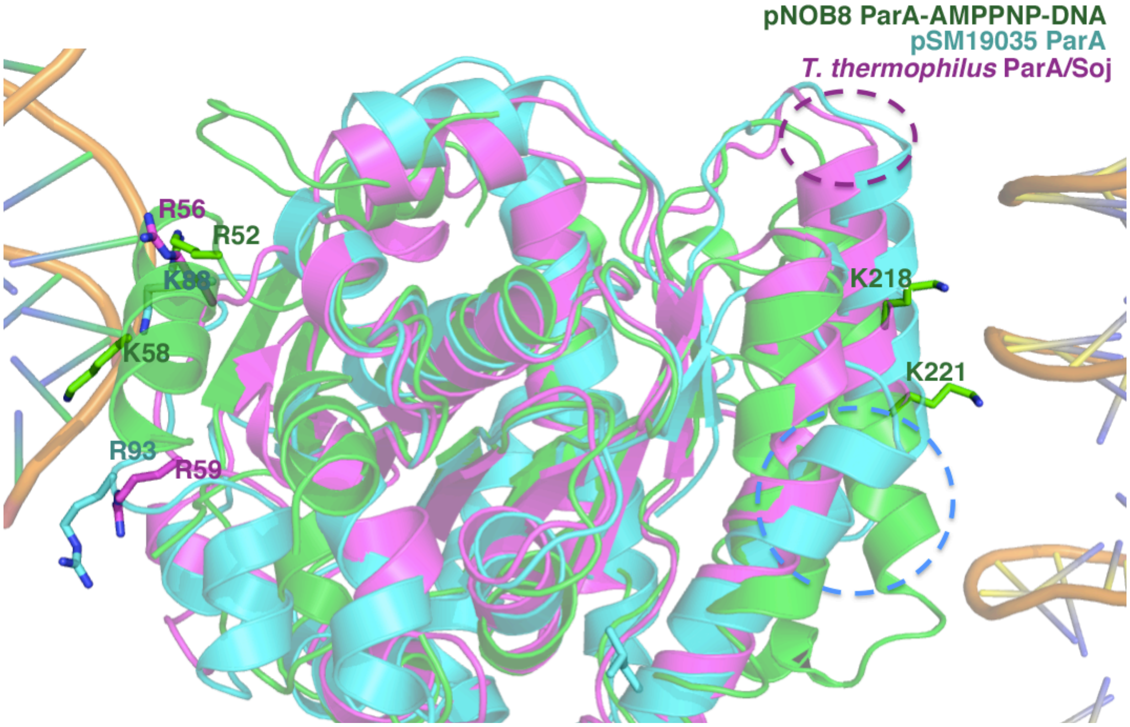

Figure S3. Superimpositions of *T. thermophilus* Soj or ParA (magenta) and pSM19035 ParA (cyan) onto the pNOB8 ParA-AMPPNP-DNA (green) structure. nsDNA binding residues in pNOB8 ParA are shown as green sticks. The location of residues implicated experimentally to be involved in DNA binding by *T. thermophilus* ParA and pSM19035 ParA proteins are encircled in magenta and cyan. These regions are near pNOB8 ParA DNA binding residues Lys221 and Arg218. Also shown are other key pNOB8 ParA DNA binding residues, Arg52 and Lys58. Notably, both *T. thermophilus* and pSM19035 ParA proteins have similarly placed basic residues as pNOB8 ParA residues Arg52 and Lys58 (shown as sticks), which could contact the DNA with slight rotations.

Figure S4

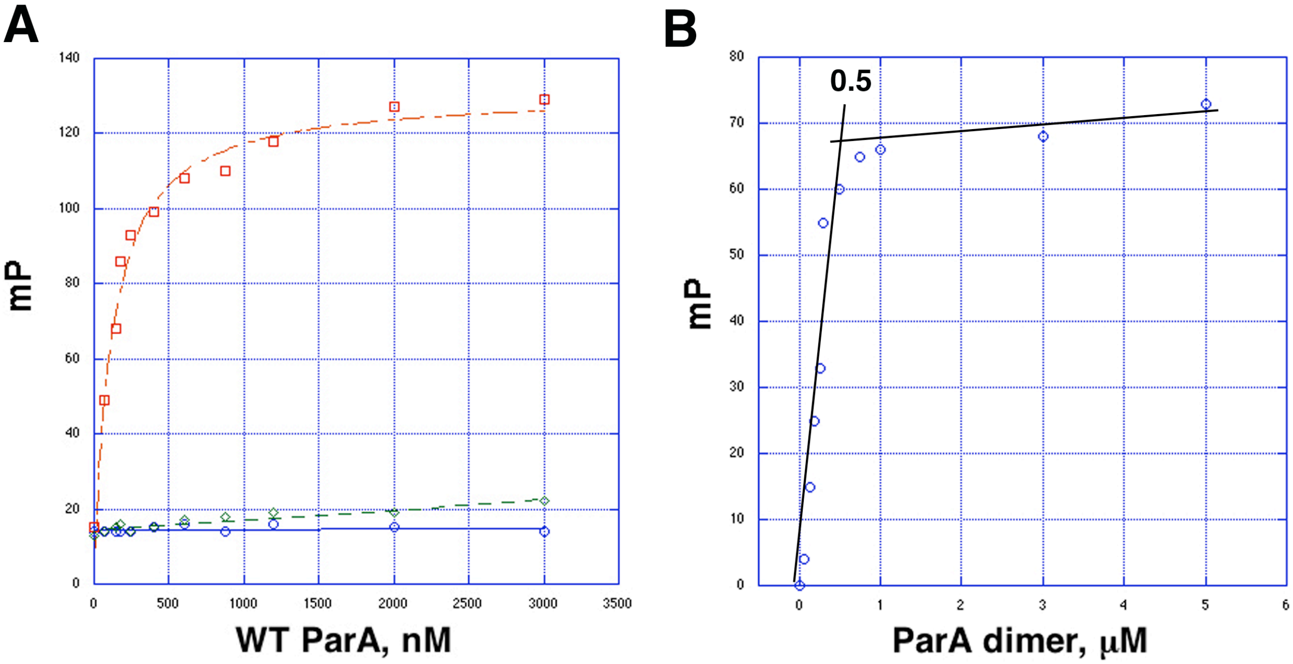

Figure S4. FP binding isotherms analyzing WT pNOB8 ParA binding to DNA and stoichiometry of binding. (***A***) WT ParA binds nsDNA in the presence of AMPPNP with a K_d of 116 ± 20 nM (red squares and red line). No binding was observed in the absence of AMPPNP (blue circles and blue line) or with ADP (green diamonds and green dashed line). The x-axis and y-axis indicate ParA concentration and millipolarization (mP) units, respectively. (***B***) To determine the ParA:DNA binding stoichiometry, the buffer and conditions were identical to those used in the FP binding affinity determination experiments in *A* except that the concentration of DNA was increased to 1 μM, which is ~10 fold above the K_d (by using a solution containing 1 nM F-DNA and 0.999 μM non fluorseceniated DNA) thereby ensuring stoichiometric binding. WT pNOB8 ParA was titrated into the binding solution and the graph of the resulting data revealed a linear increase in the observed mP until saturation, after which the mP values showed no increase. The inflection point occurs at a ParA monomer concentration of ~0.5 μM, which, when divided by the concentration of DNA (1 μM), indicates a stoichiometry of ~1 ParA dimer to 2 DNA 14mer duplexes, which supports the stoichiometry observed in the crystal structure.

Figure S5

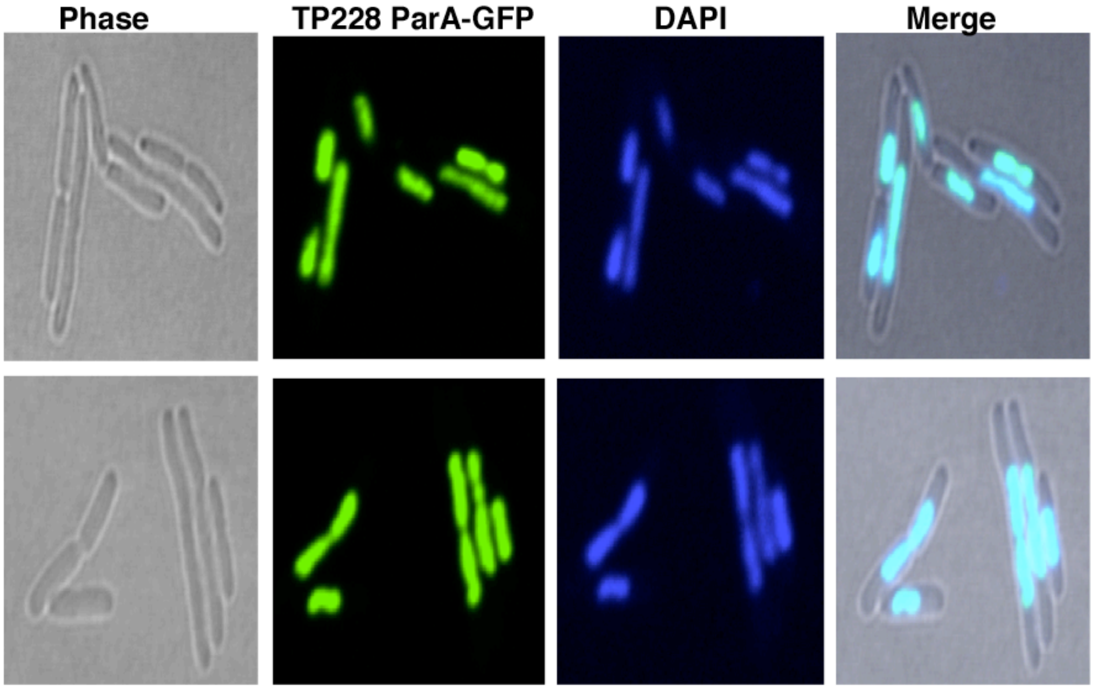

Figure S5. Confocal microscopy examining the localization of TP228 ParA-GFP and DNA (stained with DAPI). The rightmost panel, which merges GFP and DAPI images, reveal that the TP228 ParA protein colocalized with the nucleoid. Images on left were obtained from the same cells via phase contrast microscopy.

Figure S6

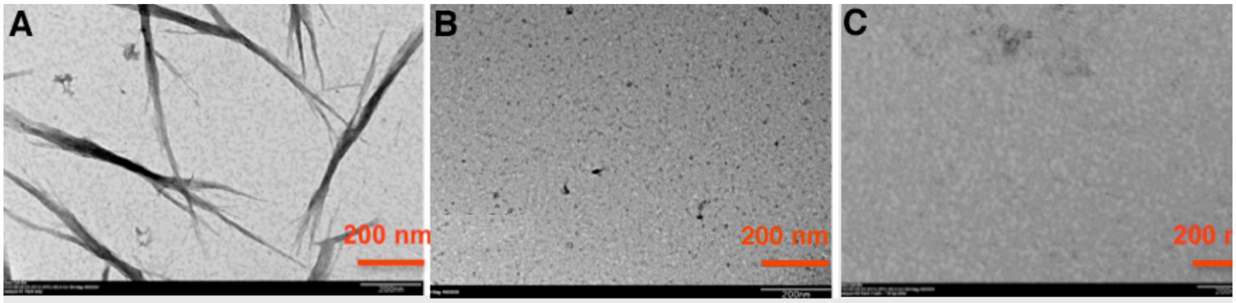

Figure S**6**. Electron microscopy (EM) analyses on pNOB8 ParA; ParA-AMPPNP-DNA does not form polymers. **(*A*)** Negative stain EM image of ParA (5 μM) in the presence of 2 mM AMPPNP and 1 mM $MgCl_2$. **(*B*)** EM image of ParA (5 μM) in the presence of 2 mM ADP and 1 mM $MgCl_2$. **(*C*)** EM image of ParA (5 μM) with 2 mM AMPPNP, 1 mM $MgCl_2$ and 10 μM DNA.

Figure S7

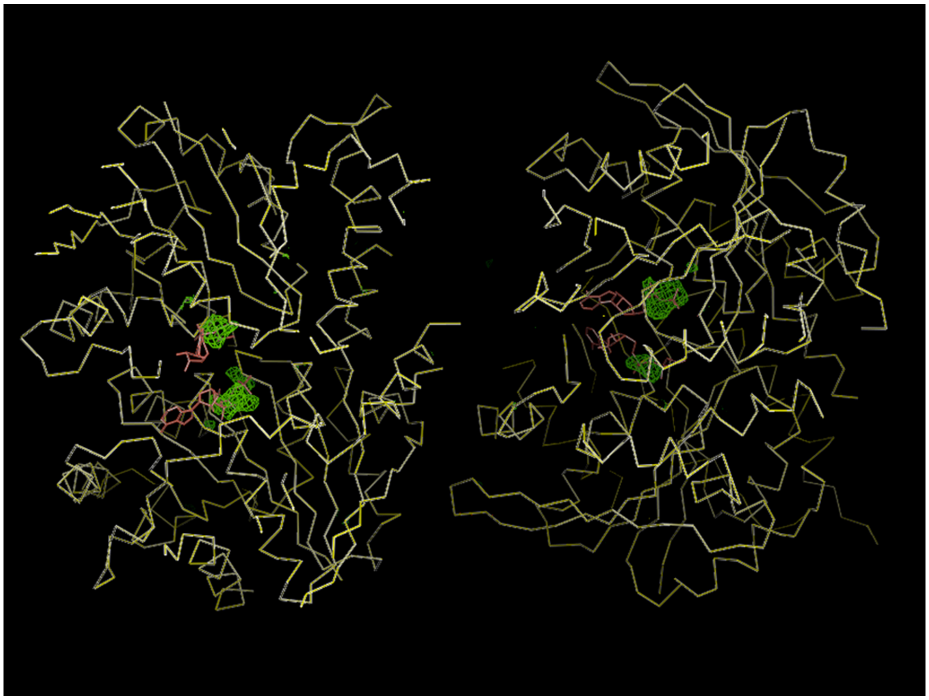

Figure S7. F_o-F_c electron density map (green mesh) contoured at 3.9 σ calculated before addition of AMPPNP molecules to the ParA-DNA structure. The map covers the two ParA dimers in the ASU. For clarity only the Cα backbone of the ParA molecules are shown and the DNA was not included. The AMPPNP molecules are shown as pink sticks.
